# TAT Modification of Alpha-Helical Anticancer Peptides to Improve Specificity and Efficacy

**DOI:** 10.1371/journal.pone.0138911

**Published:** 2015-09-25

**Authors:** Xueyu Hao, Qiuyan Yan, Jing Zhao, Wenren Wang, Yibing Huang, Yuxin Chen

**Affiliations:** 1 Key Laboratory for Molecular Enzymology and Engineering of the Ministry of Education, Jilin University, Changchun, China; 2 School of Life Sciences, Jilin University, Changchun, China; 3 National Engineering Laboratory for AIDS Vaccine, Jilin University, Changchun, China; 4 Changchun ProteLight Pharmaceutical & Biotechnology Co., Ltd., Changchun, China; Mie University Graduate School of Medicine, JAPAN

## Abstract

HPRP-A1 is an amphipathic α-helical anticancer peptide (ACP) derived from the N-terminus of ribosomal protein L1 (RpL1) of *Helicobacter pylori*. In our previously study, HPRP-A1 has been reported that induced HeLa cell apoptosis in a caspase-dependent approach and involved both by the death receptor ‘extrinsic’ pathway and the mitochondria ‘intrinsic’ pathway. Here we report the construction of a new hybrid peptide, HPRP-A1-TAT, comprising the cell-permeating peptide TAT linked to the C-terminus of HPRP-A1. This peptide exhibits higher anticancer activity against HeLa cells with lower toxicity against human RBC than HPRP-A1. Two FITC-labeled peptides, FITC-HPRP-A1 and FITC-HPRP-A1-TAT, were used to investigate and compare the cellular uptake mechanism using fluorescence spectra and flow cytometry. Compared with HPRP-A1, HPRP-A1-TAT quickly crossed cell, entered the cytoplasm via endocytosis, and disrupted the cell membrane integrity. HPRP-A1-TAT exhibited stronger anticancer activity than HPRP-A1 at the same concentration by increasing early apoptosis of HeLa cells and inducing caspase activity. Notably, after 24 h, the cellular concentration of HPRP-A1-TAT was higher than that of HPRP-A1. This result suggests that TAT protects HPRP-A1 against degradation, likely due to its high number of positively charged amino acids or the further release of peptides into cancer cells from endocytotic vesicles. We believe that this TAT modification approach may provide an effective new strategy for improving the therapeutic index and anticancer activity of ACPs for clinical use.

## Introduction

Cancer has become one of the leading causes of death in both developed and developing countries. The low efficacy and high rate of side effects of conventional therapeutic strategies, including surgery and chemotherapy, necessitates the exploration of new therapeutic drugs and strategies. Anticancer peptides (ACPs) have attracted widespread attention as a new class of anticancer drug candidates due to their high activity and unique mechanism of targeting cells [1]. Although the exact mechanism of action of ACP is unclear, most ACPs appear to affect multiple cellular processes, including necrosis, apoptosis, and gene expression, to kill cancer cells [2].

Cell-penetrating peptides (CPPs) are a family of peptides that are effectively taken up by cells [3, 4]. In 1988, Frankel and Pabo reported the first CPP, demonstrating that TAT protein from HIV-1 could pass through the cell membrane to enter cells [5, 6]. The shortest TAT peptide sequence that would still allow efficient translocation through the plasma membrane was identified [6, 7]. Since then, many CPPs, such as penetratin, R8, and BR2, have been identified [8]. These peptides are now popular transport tools in the drug delivery field because of their high transport efficiency [9] and low toxicity against normal human cells [6]. One example is the release of camptothecin inside cancer cells from a hybrid protein comprising the N-terminus of the cell-penetrating TAT and camptothecin [10]. In addition, a TAT-hybrid peptide containing the LXXLL motif was shown to be effective in treating estrogen receptor-negative tumors [11]. The uptake of albumin-based lyophilisomes by HeLa cells was increased modified by a TAT modification [12]. The design of TATp-bearing paclitaxel-loaded micelles increased the toxicity of paclitaxel against cancer cells [13]. CPPs can become cytotoxic upon retro-inversion to improve stability [14]. CPP-conjugated modifications provide a tool for transferring biologically active compounds into cells [15, 16]. In particular, the targeted delivery of CPP-conjugated drugs is a novel strategy for cancer treatment.

This study aims to explore the anticancer mechanisms of the amphipathic, α-helical ACP HPRP-A1. This peptide derived from the N-terminus of ribosomal protein L1 (RpL1) of *Helicobacter pylori*, was combined with the TAT peptide to create the hybrid peptide HPRP-A1-TAT. Using two FITC-labeled peptides (FITC-HPRP-A1 and FITC-HPRP-A1-TAT), we studied the interaction of the peptides with the cell membrane, the uptake mechanism, and the triggering of apoptosis and related enzyme activity to investigate the potential of TAT-modified peptides as anticancer therapeutics.

## Materials and Methods

### Peptide design and synthesis

HPRP-A1 (Ac-FKKLKKLFSKLWNWK-amide), HPRP-A1-TAT (Ac-FKKLKKLFSKLWNWK-RKKRRQRRR-amide), and TAT [49–57] (Ac-RKKRRQRRR-amide) peptides were synthesized using solid-phase methods by 9-fluorenyl-methoxycarbonyl (Fmoc) chemistry as described previously [17] and purified by reversed-phase high-performance liquid chromatography. FITC was conjugated to the N-terminus of HPRP-A1 and HPRP-A1-TAT through a Fmoc-ε-Ahx-OH linker using the same method. The peptides were further characterized by mass spectrometry and amino acid analysis.

### Circular dichroism spectroscopy

Circular dichroism spectra were measured using a 0.02-cm path length quartz cuvette on a Jasco J-810 spectropolarimeter (Jasco, Tokyo, Japan) at 25°C as described previously [17]. HPRP-A1 and HPRP-A1-TAT peptides were dissolved at pH 7.4 and 25°C in 50 mM KH_2_PO_4_/K_2_HPO_4_ containing 100 mM KCl without TFE or in the presence of 50% TFE. The mean residue molar ellipticities were calculated using the equation [*θ*] = *θ*/10*lcMn*, where *θ* is the ellipticity in millidegrees, *l* is the optical path length of the cuvette in centimeters, *cM* is the peptide concentration in mole/liter, and *n* is the number of residues in the peptide.

### Cell cultures and cell viability assays

Human cervical carcinoma cells (HeLa), human gastric carcinoma cells (SGC-7901), human hepatocellular carcinoma cells (HepG2), and mouse melanoma cells (B16) were obtained from the American Type Culture Collection. All cells were cultured in DMEM medium containing 100 U/ml penicillin, 100 mg/ml streptomycin, and 10% fetal bovine serum at 37°C with 5% CO_2_.

The activities of HPRP-A1, HPRP-A1-TAT, and TAT against cancer cells were evaluated using MTT cell assay. Cells (5 × 10^3^) were seeded into 96-well plates and incubated with serially two-fold diluted concentrations of different peptides (0.5–250 μM) for 24 or 1 h at 37°C. As a negative control, cells were cultured without the addition of the peptides. After incubation, 20 μl/well of MTT solution (5 mg/ml) was added to all test wells, and the cells were treated for 4 h at 37°C. Dimethyl sulfoxide (150 μl/well) was added before spectrometric determination at 492 nm using a microplate reader (GF-M3000; Gaomi Caihong Analytical Instruments Co., Ltd. Shandong, China). The results were expressed as anticancer activity (IC_50_), the concentration at which cell viability was inhibited by 50% compared with control cells. The MTT assays were repeated in triplicate.

### Hemolytic activity

As previously described [17], peptides were serially diluted in PBS in round-bottomed 96-well plates to give a volume of 70 μl sample solution/well. After incubation for 24 or 1 h, hemolytic activity was determined as the minimal peptide concentration to cause hemolysis (minimal hemolytic concentration, MHC). Erythrocytes in PBS and distilled water were used as negative (0%) and positive (100%) hemolysis controls, respectively.

### Confocal microscopy

Images of cells were obtained by laser scanning confocal microscope (LSM710, Carl Zeiss, Oberkochen, Germany). Briefly, HeLa cells (4 × 10^5^) were cultured in six-well plates. After overnight culture, the cells were washed with PBS three times and then incubated with FITC-labeled HPRP-A1 and HPRP-A1-TAT after staining with 4,6-diamidino-2-phenylindile (blue) for 4 h at 37°C. Images of cells (400× magnification) were captured every 30 s from 0 to 180 s. The concentrations of each peptide were 2, 4, and 8 μM.

### Lactate dehydrogenase leakage assay

The lactate dehydrogenase (LDH) release assay was used to determine the extent of membrane permeability [10, 18, 19]. Briefly, HeLa cells (1 × 10^4^) were seeded in 96-well plate for 24 h and then incubated with 100 μl of serum-free medium containing 2, 4, or 8 μM HPRP-A1, HPRP-A1-TAT, or TAT for 1 h. Untreated cells were used as a control. Cells incubated with 1% triton X-100 served as the positive control. Data were measured at 450 nm. Untreated cells were taken as no leakage, and 100% leakage was defined as total LDH release.

### Flow cytometry analyses

To explore the relationships between the cellular uptake of peptides and ATPs, HeLa cells were placed at 4°C for 1 h to consume the intracellular ATPs before incubating with the peptides of FITC-HPRP-A1 and FITC-HPRP-A1-TAT. Briefly, for assays at 4°C, cells were maintained in a customer-built cooling chamber while cells without cooling as the control. After 1 h, the peptides with different concentrations were added to cells and incubated for 1 h, then fluorescence analysis was performed using flow cytometry uptake expressed as the median of cell fluorescence distribution (normalized to the cell fluorescence distribution median in untreated control cells at 37°C) [20].

Cell apoptosis was detected by flow cytometry (FACSCalibur, Becton-Dickinson, San Jose, CA, USA). Briefly, HeLa cells (1 × 10^6^) were seeded in six-well plates. One day later, HPRP-A1 and HPRP-A1-TAT (2, 4, or 8 μM) was added to each well for 1 and 24 h. Cells were then collected and analyzed.

The degradation of internalized FITC-HPRP-A1 and FITC-HPRP-A1-TAT peptides in cells was also detected using flow cytometry. HeLa cells (1 × 10^6^) were cultured in six-well plates for 24 h, and peptides were then added to each well for 1 and 24 h after washing three times with PBS. Fluorescence analysis was performed using flow cytometry. Untreated cells were used as controls.

### Apoptosis assay

Apoptosis of HeLa cells was detected using the Annexin V-FITC apoptosis detection kit. The mitochondrial membrane potential was detected using the 5,5′,6,6′-tetrachloro-1,1′,3,3′-tetraethylbenzimidazolcarbocyanine iodide (JC-1) detection kit, and the activity of caspase-3, -8, and -9 was tested using the corresponding caspase activity detection kits according to the manufacturers’ instructions. HeLa Cells were treated with HPRP-A1 and HPRP-A1-TAT at concentrations of 2, 4, or 8 μM. All detection kits were purchased from Bestbio, Shanghai, China.

## Results

### Peptide design and characterization

As described previously, HPRP-A1 is an α-helical amphipathic membrane-active peptide consisting of 15 amino acids with good anticancer activity [17]. Derived from the N-terminus of RpL1 of *H*. *pylori*, this peptide has greater than 86% sequence homology with the original sequence and exhibits an α-helical structure in a hydrophobic environment [21]. In this study, a CPP of TAT was conjugated to the carboxyl terminus of HPRP-A1 to form a hybrid peptide of HPRP-A1-TAT, and the transmembrane potential and the mechanism of action of HPRP-A1 were explored. In addition, FITC-HPRP-A1 and FITC-HPRP-A1-TAT were used to investigate the anticancer mechanism.

**Fig 1:**
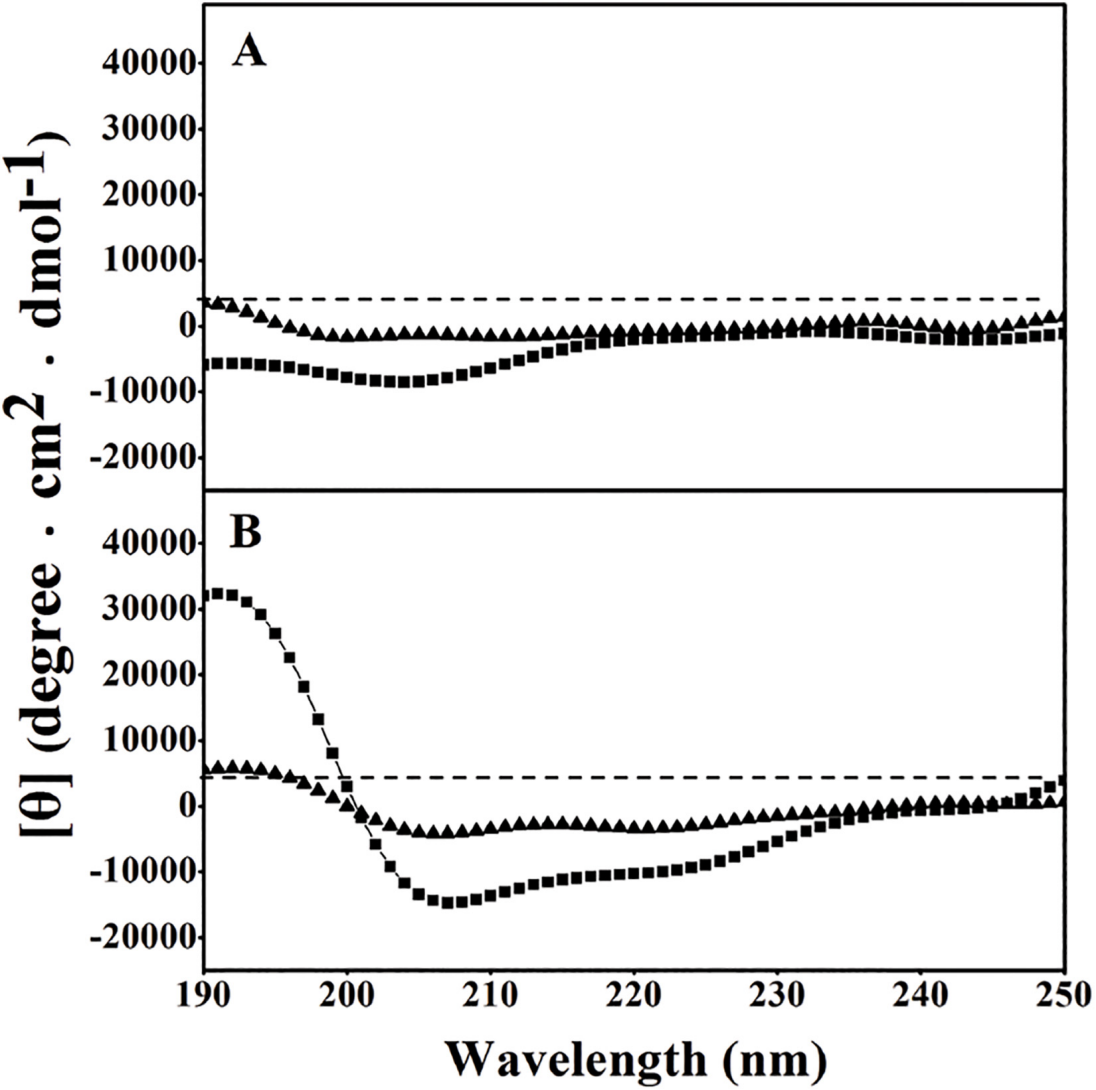
Circular dichroism spectra of peptides. (**A**) In benign medium (50 mM KH_2_PO_4_/K_2_HPO_4_ containing 100 mM KCl, pH 7.4) at 25°C and (**B**) in the presence of 50% TFE at 25°C. The symbols used are as follows: ■, HPRP-A1 peptide; ▲, HPRP-A1-TAT peptide.

The sequences and the relative hydrophobicity of peptides are shown in Table 1. From Table 1, it is clear that the hydrophobicity of HPRP-A1-TAT is remarkably lower than that of HPRP-A1. This feature may be attributed to the high hydrophilicity of TAT, with its high number of charged amino acids. As shown in Fig 1, the secondary structures of the peptides were measured in benign conditions (50 mM KH_2_PO_4_/K_2_HPO_4_ containing 100 mM KCl, pH 7.4, mimicking the hydrophilic environment) and in the presence of 50% TFE (mimicking the hydrophobic environment) by circular dichroism spectroscopy. HPRP-A1 and HPRP-A1-TAT both exhibited a random coil structure under benign conditions. Although both peptides exhibited an α-helical structure under hydrophobic conditions (50% TFE), the helical content of HPRP-A1-TAT was significantly lower than that of HPRP-A1, indicating the importance of peptide hydrophobicity in maintaining the α-helical structure.

**Table 1:** Sequences and biophysical data of peptides used in the study.

Peptide	Amino acid sequence	Mw	*t*_R_ (min)^a^
TAT	Ac-RKKRRQRRR-amide	1380.66	13
HPRP-A1	Ac-FKKLKKLFSKLWNWK-amide	2035.53	41
HPRP-A1-TAT	Ac-FKKLKKLFSKLWNWKRKKRRQRRR-amide	3357.13	31.5

^a^ *t*_R_ (min) denotes the retention time at 25°C by reversed-phase HPLC.

### Anticancer and hemolytic activities

The anticancer activities of HPRP-A1 and HPRP-A1-TAT against four different cancer cell lines are shown in Table 2. For all cell lines tested, HPRP-A1-TAT exhibited stronger anticancer activity than HPRP-A1. HPRP-A1-TAT exhibited significantly lower toxicity against human RBC than HPRP-A1, with hemolytic activity >500 and 64 μM, respectively. Therapeutic index values are used to evaluate the specificity of peptides against cancer cells and are calculated as the ratio of the MHC to the IC_50_. The larger the therapeutic index, the greater the anticancer specificity [17, 22]. As shown in Table 2, the therapeutic indices of HPRP-A1 and HPRP-A1-TAT are 8.6 and 256, respectively, indicating that the specificity of HPRP-A1-TAT against cancer cells is approximately 30-fold greater than that of HPRP-A1.

**Table 2:** Anticancer (IC_50_) and hemolytic activities (MHC) of peptides against cancer cells and human red blood cells.

Peptide	IC_50_ ^a^ (μM)						MHC^c^ (μM)	Therapeutic index^d^
	24 h					1 h		
	B16	SGC-7901	HepG2	HeLa	GM^b^	HeLa		
HPRP-A1	6.1 ±0.02	5.2 ±0.14	7.7 ±0.23	3.5 ±0.03	5.6	7.4 ± 0.12	64 ±6.80	8.6
HPRP-A1-TAT	3.9 ±0.02	4.8 ±0.08	5.8 ±0.36	1.8 ±0.02	4.1	3.9 ± 0.07	>500	256

^a^Anticancer activity (IC_50_) represents the concentration of peptides at which cell viability was inhibited by 50% in comparison with the untreated cells. The MTT assay was repeated in triplicate, and IC_50_ value was determined by averaging three repeated experiments.

^b^GM of the anticancer activity (IC_50_) for the four cancer cell lines.

^c^Hemolytic activity (MHC) was determined using human red blood cells after incubation with peptides for 1 h. If no hemolytic activity was observed at 500 μM, a value of 1000 μM was used for calculating the therapeutic index.

^d^Therapeutic index = MHC/IC_50_. Larger values indicate greater anticancer specificity.

GM, geometric mean; MHC, minimal hemolytic concentration.

### Cellular uptake and interaction between peptides and the cell membrane

**Fig 2:**
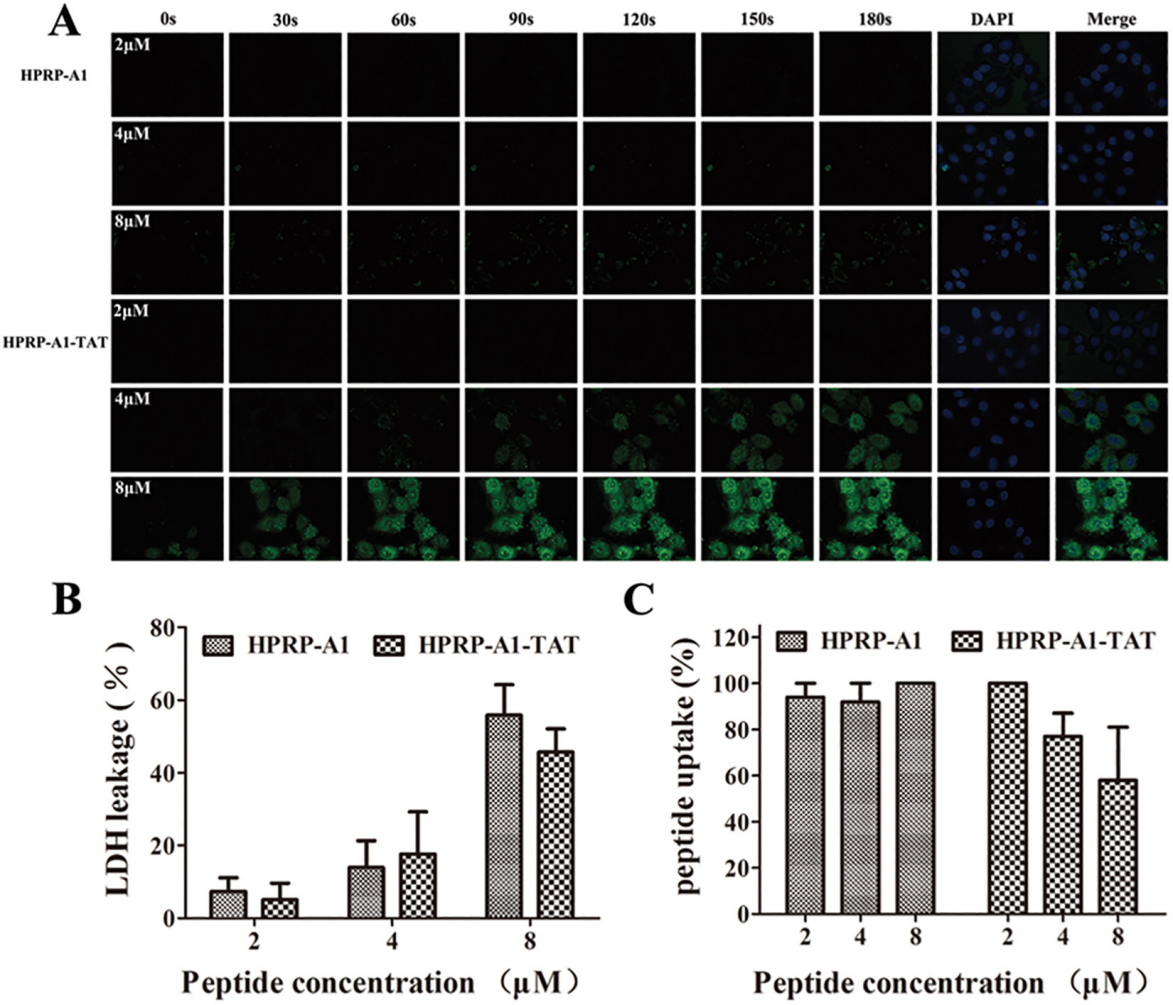
Cellular uptake and interaction between peptides and cell membranes. (**A**) The fluorescence time profiles of interaction between peptides and membranes. HeLa cells were incubated with different concentrations of FITC-labeled HPRP-A1 and HPRP-A1-TAT at concentrations of 2, 4, and 8 μM. Images (400× magnification) were captured by laser scanning confocal microscopy every 30 s from 0 to 180 s. Green, FITC peptides; blue, 4,6-diamidino-2-phenylindile-stained nuclei. (**B**) LDH leakage assay. HeLa cells were incubated with HPRP-A1 and HPRP-A1-TAT at 2, 4, and 8 μM for 1 h and LDH assayed. (**C**) Cellular uptake of peptides, measured by flow cytometry. After incubation for 1 h at 4°C, HeLa cells were incubated with FITC-HPRP-A1 or FITC-HPRP-A1-TAT peptides for 1 h. The cells were those cultured and treated with peptides at 37°C were used as controls. Data are presented as the mean ± SD of three independent experiments. LDH, lactate dehydrogenase.

FITC-HPRP-A1 and FITC-HPRP-A1-TAT peptides were used to investigate the rate of cellular uptake by laser scanning confocal microscopy, as shown in Fig 2A. To simplify the experiments, only HeLa cells were used in the following assays. For both HPRP-A1 and HPRP-A1-TAT, the rate of cellular uptake gradually increased with increasing peptide concentration. However, the rate of cellular uptake of HPRP-A1-TAT was significantly higher and the cellular uptake speed dramatically faster than those of HPRP-A1 at the same concentration. For example, at a peptide concentration of 8 μM, the fluorescence intensity of HPRP-A1-TAT at 30 s was dramatically stronger than that of HPRP-A1 at 180 s, showing the ability of the peptide to traverse the cell membrane.

To test the integrity of the cell membrane, the LDH leakage assay was performed after the HeLa cells were incubated with the HPRP-A1 and HPRP-A1-TAT peptides for 1 h. The IC_50_ values of the peptides against HeLa cells for 1 h are shown in Table 2. LDH leakage and thus cell membrane damage increased with increasing peptide concentrations (Fig 2B). However, the degree of damage between the two peptides at the same concentration did not differ. This result is consistent with previous reports that peptides with TAT have good biocompatibility [6, 8], and HPRP-A1 indeed disrupts the integrity of the cell membrane [23].

As previously reported, the TAT peptide can cross the cell membrane via energy-dependent endocytosis without specific receptors [6, 24, 25], while HPRP-A1 destroys the integrity of the cell membrane [23]. Thus, we believe that the hybrid peptide HPRP-A1-TAT crosses cell membranes via two different mechanisms, including endocytosis and damage cell membrane integrity. To further characterize the mechanism of HPRP-A1-TAT uptake, FITC-HPRP-A1 and FITC-HPRP-A1-TAT were used to determine whether ATP is needed for their cellular uptake via flow cytometry and the data are showed in Fig 2C. It is clear that the cellular uptake of HPRP-A1 was not influenced by temperature and the data differences were negligible between conditions at 37°C (with ATP) or at 4°C (without ATP), however, the cellular uptake of HPRP-A1-TAT significantly decreased after ATP depletion at 4°C in a dose-dependent manner. Based on the data of LDH, we believe that HPRP-A1-TAT may cross the cell membrane through two different processes, including membrane disruption and energy-dependent endocytosis, while HPRP-A1 crosses cell membranes only by damaging membrane integrity.

### Cell apoptosis assay

**Fig 3:**
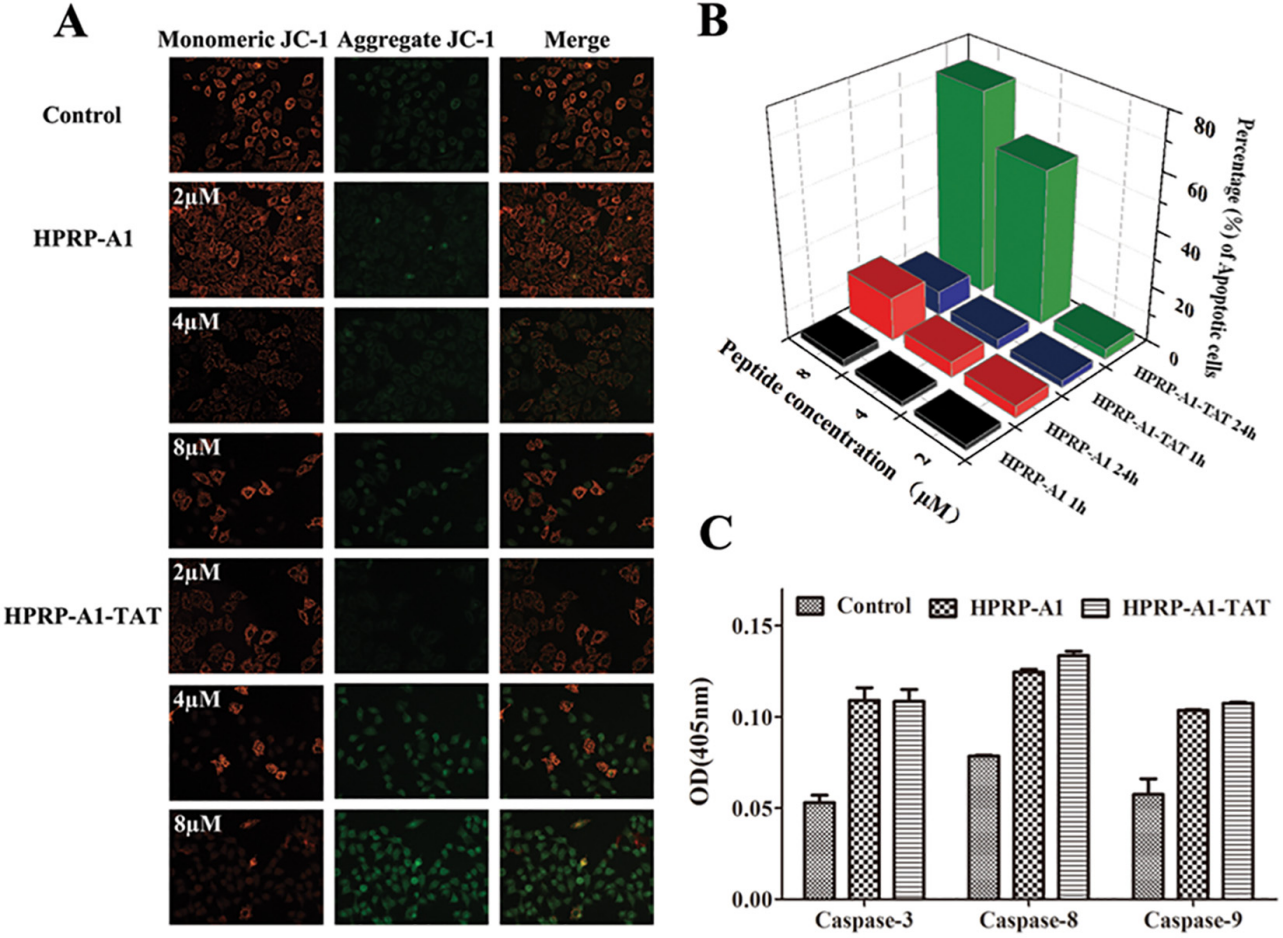
Peptide-induced cell apoptosis. (**A**) Mitochondrial membrane potential measured by JC-1, an indicator of mitochondrial function. HeLa cells were treated with various concentrations of HPRP-A1 or HPRP-A1-TAT for 24 h. (**B**) Percentage of early apoptotic cells, as assessed by flow cytometry. HeLa cells were treated with various concentrations of HPRP-A1 or HPRP-A1-TAT for 1 or 24 h. (**C**) Caspase-3, -8, and -9 activity. HeLa cells were treated with HPRP-A1 (4 μM) or HPRP-A1-TAT (4 μM) for 24 h before measuring caspase activity levels. Data are presented as the mean ± SD of three independent experiments.

HPRP-A1-TAT exhibited faster cellular uptake and stronger anticancer activity and specificity against HeLa cells than did HPRP-A1 (Table 2). To investigate the mechanism of HPRP-A1-TAT action, cell apoptosis assays were performed. Mitochondrial membrane potential was measured using JC-1, an indicator of mitochondrial function (Fig 3A). The mitochondria membrane potential decreased significantly with increasing concentrations of both the HPRP-A1 and HPRP-A1-TAT peptides. HPRP-A1-TAT treatment resulted in greater changes in the mitochondria membrane potential than did HPRP-A1 at the same concentration.

Early apoptosis of HeLa cells was examined by flow cytometry with Annexin V/PI staining. HeLa cells were treated with HPRP-A1 and HPRP-A1-TAT (2, 4, and 8 μM) for 1 and 24 h (Fig 3B). After treatment with either HPRP-A1 or HPRP-A1-TAT for 1 h, only a few HeLa cells (<10%) were apoptotic, even at high concentrations; in contrast, after 24 h of treatment with HPRP-A1-TAT, the percentage of early apoptotic cells increased significantly, reaching 55% (4 μM) and 72% (8 μM). Thus, HPRP-A1-TAT strongly induces HeLa cell apoptosis.

To further test the ability of the peptides to induce apoptosis, the caspase activities were determined (Fig 3C). The results indicate that the apoptosis-related enzymes caspase-3, -8, and -9 were activated by both HPRP-A1 and HPRP-A1-TAT. Thus, we believe that the apoptotic mechanism of HPRP-A1-TAT may involve caspase-dependent pathways in both the death receptor ‘extrinsic’ pathway and the mitochondria ‘intrinsic’ pathway in HeLa cells [23, 26].

### Degradation of internalized peptides

**Fig 4:**
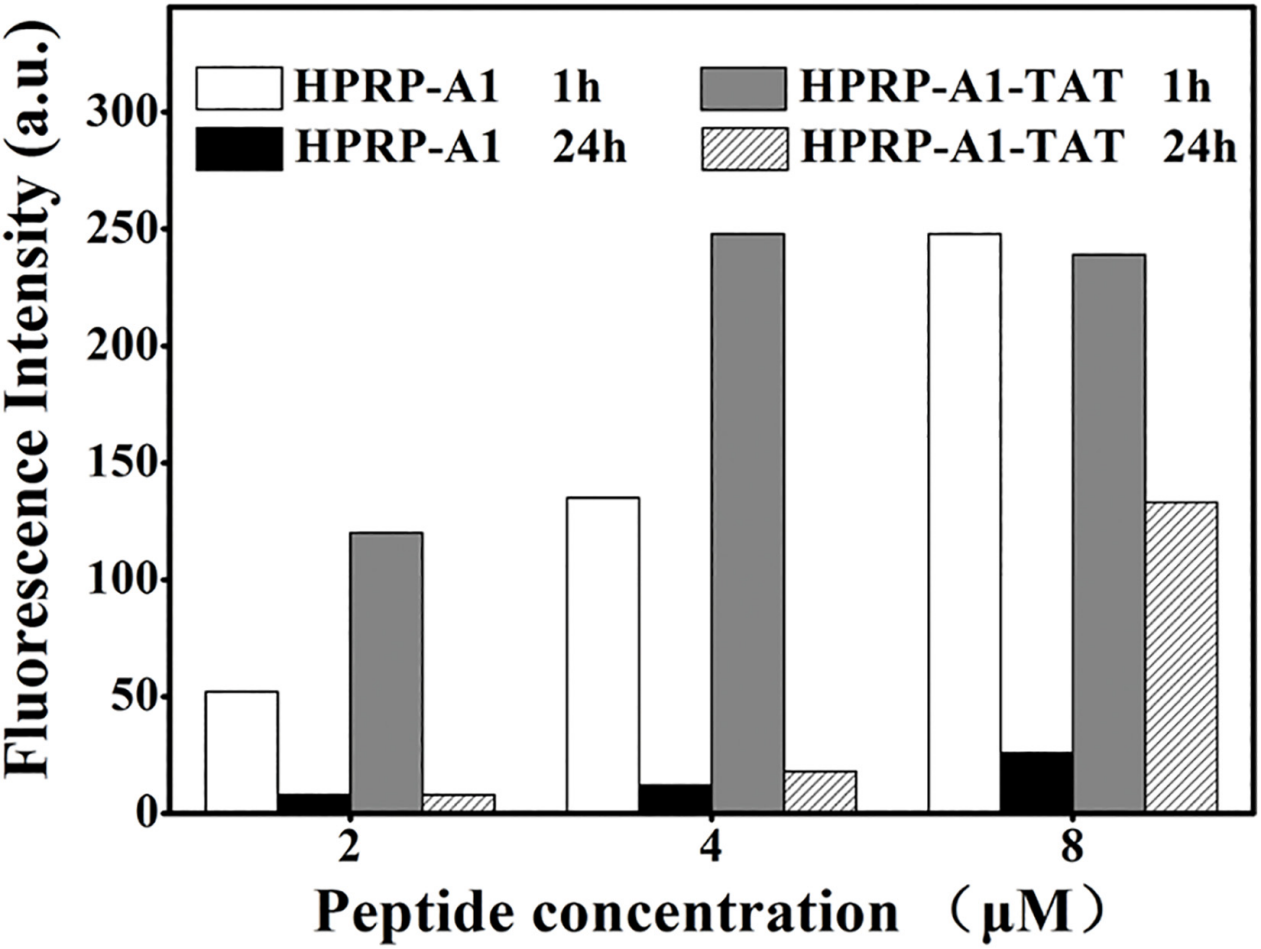
The degradation of peptides by flow cytometry analysis. HeLa cells were incubated with FITC-labeled peptides (2, 4, or 8 μM) for 1 or 24 h. Cellular uptake of peptides is expressed as the median of cell fluorescence distribution by flow cytometry.

Internalized peptides are digested by enzymes in the cytoplasm or within lysosomes. In this study, the degradation of internalized peptides of FITC-HPRP-A1 and FITC-HPRP-A1-TAT in cells was detected by flow cytometry after incubation with HeLa cells for 1 and 24 h, respectively. From the uptake of HPRP-A1-TAT was clearly faster than that of HPRP-A1 (Fig 4). The fluorescence intensity of cells treated with 4 or 8 μM FITC-HPRP-A1-TAT was similar and reached the maximum values. For FITC-HPRP-A1, the fluorescence intensity gradually increased with increasing peptide concentrations and reached the maximal value at 8 μM. At the lower concentrations of both peptides (2 and 4 μM), significantly peptide digestion was observed after incubation for 24 h. In contrast, at the higher peptide concentration (8 μM), fluorescence intensity after 24 h incubation was much higher in cells treated with FITC-HPRP-A1-TAT than in those treated with FITC-HPRP-A1, indicating that there were more HPRP-A1-TAT molecules kept inside the cells.

## Discussion

Peptide-based transport systems, such as CPPs, have become a popular transport tool in drug delivery field due to its high efficiency and low toxicity. In this study, a typical CPP, TAT was hybridized to the membrane-active ACP HPRP-A1. The cellular uptake process and anticancer activity and mechanism of HPRP-A1-TAT were explored.

Hydrophobicity and helicity are the key parameters determining the biological activity of membrane-active peptides [17, 27, 28]. To improve the specificity of a given peptide, the hydrophobicity and helicity should be kept within a reasonable range. The hydrophobicity and helicity of HPRP-A1-TAT were markedly lower than those of HPRP-A1 (Fig 1 and Table 1). However, the therapeutic index of HPRP-A1-TAT was significantly higher than that of HPRP-A1, as indicated by its higher anticancer activity and lower toxicity (Table 2).

The proposed mechanism of action of membrane-active ACPs involves peptide targeting of the cell membrane and destruction of cell membrane integrity driven by strong electrostatic and hydrophobic interactions [2, 17, 29, 30]. While the membranes of normal cells, such as RBC, are characterized by zwitterionic phospholipids, many cancer cell membranes have more anionic phospholipids in their outer leaflet and a higher number of microvilli [31]. The anticancer specificity of peptides depends on this compositional difference between normal and cancer cell membranes. Compared with the peptide HPRP-A1, HPRP-A1-TAT has more positive charges and may have more chances to interact with the anionic surface of cancer cells [25, 31, 32]. In addition, HPRP-A1-TAT may enter the cytoplasm via two pathways—endocytosis and disruption of cell membrane integrity. TAT may increase the rate of cellular uptake of HPRP-A1-TAT, while HPRP-A1 only enter the cytoplasm via disruption of cell membrane integrity, thus HPRP-A1-TAT exhibited higher anticancer activity than HPRP-A1. However, the lower hydrophobicity and helicity of HPRP-A1-TAT result in weak hydrophobic interactions with cell membranes; hence, HPRP-A1-TAT exhibited lower toxicity against normal human cells than did HPRP-A1.

Laser scanning confocal microscopy indicated that HPRP-A1-TAT crosses the cell membrane faster than HPRP-A1 (Fig 2A). LDH release assays were performed to reflect the extent of membrane permeability [10], indicating no significant difference in membrane between cells treated with HPRP-A1 and HPRP-A1-TAT at the same concentrations (Fig 2B). Previous studies have reported TAT cellular uptake to involve endocytosis and heparin sulfate receptors [32] as well as interaction with ions on the cell membrane surface [33]. Molecular dynamics simulation of the interaction between the membrane phospholipid bilayer and TAT also indicated that the main mode of TAT uptake was endocytosis [34]. In this study, HeLa cells treated with TAT showed no detectable release of LDH, confirming the previous report that TAT enters the cells via endocytosis.

Endocytosis is an energy-dependent process; low temperature inhibits ATP production and then affects endocytosis [20]. ATP depletion only inhibited the endocytosis of HPRP-A1-TAT but not HPRP-A1 (Fig 2C). Confocal microscopy images also show that the HeLa cell surface was not smooth after treatment with 8 μM HPRP-A1-TAT (data not shown). Hence, it seems that HPRP-A1-TAT crosses cell membranes by damaging membrane integrity and through energy-dependent endocytosis, while HPRP-A1 crosses cell membranes just by damaging the membrane integrity.

Mitochondrial apoptosis is believed to play a crucial role in effective cancer treatment [35]. In this study, we observed that HPRP-A1-TAT not only enters cancer cells but also induces cell apoptosis by activating caspase-3, -8, and -9 (Fig 3). Cells treated with HPRP-A1-TAT exhibited a concentration-dependent decrease in mitochondrial membrane potential compared with those treated with HPRP-A1. Flow cytometry indicated that HPRP-A1-TAT induced more cells to enter early apoptosis than did HPRP-A1, which is consistent with the mitochondrial membrane potential results.

The mitochondrial ‘intrinsic’ pathway is one of the principal pathways leading to apoptosis [36]. At the beginning of mitochondrial cell death pathway, the permeability of the mitochondrial membrane was increased, which resulted to the release of various apoptotic factors into the cytoplasm and induced cell apoptosis. Various cationic peptides have been demonstrated to selectively disrupt mitochondrial membranes due to the different membrane potentials between mitochondrial membrane and plasma membrane [35], such as when the cells were treated with BRBP1-TAT-KLA, an anticancer peptide, mitochondrial damages appeared as swollen vesicle-like structures [37]. In this study, compared to the peptide of HPRP-A1, HPRP-A1-TAT has more chances to interact with mitochondrial membrane and destroy the integrity of the membrane due to more positive charges. Interestingly, the change of mitochondrial membrane permeability can cause a decrease of the mitochondrial membrane potential, which is consistent with our results that HPRP-A1-TAT treatment resulted in greater changes in the mitochondria membrane potential than did HPRP-A1 at the same concentration.

Decreasing the rate of therapeutic peptide degradation is a challenging problem [38]. Previous studies have shown that TAT-conjugated peptides were degraded in the lysosome [20]. However, from Fig 4, it is clear that the degradation of 8 μM HPRP-A1-TAT was slower than that of 8 μM HPRP-A1 after 24 h of treatment. This result suggests that TAT protects HPRP-A1 against degradation, likely due to its high number of positively charged amino acids or the release of further peptides into cancer cells from endocytotic vesicles. In contrast, HPRP-A1 might be digested in lysosomes, since it crosses cell membranes only through disrupting cell membrane integrity. In addition, as shown in Figs 2A and 4, TAT-modified peptide could enter into HeLa cells faster and more in amount than HPRP-A1 at the same concentration; furthermore, more HPRP-A1-TAT molecules kept inside the cells after 24h. These results indicated that HPRP-A1-TAT exhibited anticancer activity not only with a rapid effect, but also with a continuous process.

In summary, TAT modification of the ACP HPRP-A1 increases its rate and efficiency of cellular uptake, increases its anticancer activity, decreases its cell toxicity, and results in higher specificity against cancer cells. Furthermore, the new hybrid peptide HPRP-A1-TAT has been proved to cross cell membranes via two different routes: disruption of cell membrane integrity and energy-dependent endocytosis, thereby inducing further apoptosis in cancer cells. We believe that this TAT-modification approach may provide an effective new strategy for improving the therapeutic index and anticancer activity of ACPs for clinical use.
